# Growth and gut performance of young pigs in response to different dietary cellulose concentration and rearing condition

**DOI:** 10.5713/ab.20.0721

**Published:** 2021-01-01

**Authors:** Hyun Min Cho, Eunjoo Kim, Samiru Sudharaka Wickramasuriya, Taeg Kyun Shin, Jung Min Heo

**Affiliations:** 1 Department of Animal Science and Biotechnology, Chungnam National University, Daejeon 34134, Korea

**Keywords:** Cellulose, Dietary Fiber, Digestibility, Environmental Conditions, Intestinal Health

## Abstract

**Objective:**

This experiment was conducted to investigate the effect of insoluble cellulose supplementation to diets on the growth performance, intestinal morphology, the incidence of diarrhea, nutrients digestibility, and inflammatory responses in altering environmental conditions of animals housing.

**Methods:**

A total of 108 male pigs (Duroc×[Yorkshire×Landrace]) were randomly allocated to one of three dietary treatments (cellulose 0%, 1%, 2%) and two environmental conditions (good sanitary condition vs. poor sanitary condition) to give 6 replicate pens per treatment with three pigs per each pen at 14 days post-weaning.

**Results:**

Pigs were in good sanitary condition had higher average daily gain (p<0.01) and improved feed efficiency (p<0.05) from day 1 to 14 after weaning compared to their counterparts. The interactions were found between environmental conditions and dietary treatments (day 7: crypt depth [p<0.01], villous height to crypt depth [p<0.001]; day 14: crypt depth [p<0.001], villous to crypt ratio [p<0.01]) in ileum morphology. Crypt depth was decreased (p<0.05), and villous to crypt ratio was increased (p<0.05) only in poor sanitary conditions. Pigs exposed to the good sanitary condition had higher (p<0.05) apparent ileal digestibility (day 7, gross energy; day 14, dry matter), apparent total tract digestibility (day 14, dry matter and crude protein) compared to pigs housed in the poor sanitary condition. Meanwhile, pigs fed a diet supplemented with 2% cellulose had decreased (p<0.05) apparent ileal digestibility (day 7, dry matter; day 14, crude protein), apparent total tract digestibility (day 7, dry matter; day 14, crude protein, gross energy) compared to pigs fed a diet supplemented with 0% or 1% cellulose.

**Conclusion:**

Our results indicated that a diet supplemented with 1% cellulose increased villous to crypt ratio, however feeding a diet containing cellulose (1% or 2%) impaired nutrient digestibility for 14 day after weaning in both good sanitary and poor sanitary conditions.

## INTRODUCTION

Weaning pigs inevitably encounter multifactorial stressors including psychologic (e.g., new facility and littermates), physiologic (e.g., separation from mother), and metabolic (e.g., change of intestinal morphology and physical diet form) complications [1]. One such challenge is impaired growth rate due to post-weaning diarrhea during the first day 5 to 11 after weaning, which causes immune dysfunction, immature enzyme activities of the gastrointestinal tract and gut microflora, and decreased growth performance of pigs [1]. Intestinal features should be well-developed in the weaning pigs to cope with multifactorial stressors to maintain healthy guts and should have remained intact as the gastrointestinal tract is the largest surface that encounters the external materials (i.e., feed). Moreover, the gut microbiota that is mainly located in the hindgut of the weaning pigs must be well-balanced [2], and provides gross energy (GE) and the nutrients and regulates the mucosal immune system by educating the naive infant immune components and serves as a source of immune stimulators [3]. Dietary fiber has a great influence on the overall health status of young pigs [4]. Most plant-origin feed ingredients contain considerable amounts of dietary fiber, which are categorized as soluble and insoluble dietary fibers based on their solubility [5]. As we all known, the soluble dietary fiber can be easier fermented in the hindgut, thus producing more short chain fatty acids than insoluble dietary fiber [5]. Although most of the complex fraction of insoluble dietary fiber (cellulose) is not depolymerized by the endogenous enzymes of non-ruminant animals, insoluble dietary fiber provides health benefits by serving as the main substrate for the intestinal microbiota to produce volatile fatty acids that can be GE contributors to the host animals [6]. Moreover, insoluble dietary fiber can positively stimulate the intestinal pathway to boost the digesta flow rate by washing mucin at the gut lumen, and subsequently, playing an abrasive role by irritating the mucin secretion of goblet cells [7]. It is well-known that i) if the improvement in the mucin synthesis capacity is prominent, then higher gastrointestinal protection of bacterial invasion is expected, ii) insoluble dietary fiber acts as a prebiotic that regulates the interaction between mucus and microorganisms, controlling the gut environment and the immune system [8], and iii) extruded maize or rice-based diets that include a moderate level of insoluble dietary fiber can decrease the incidence of diarrhea in weaning pigs [9].

In general, growth performance and gut health-related experiments with weaning pigs have been performed in good sanitary environments rather than in typical commercial farm environments [1]. Yet, the poor sanitary environment present in farm-like conditions can critically influence gut health together with growth during the weaning period [10]. Floc’h et al [11] suggested that these poor sanitary conditions attenuated the inflammatory response and consequently increased an adverse effect on growth performance after weaning. In addition, a poor sanitary environment interfered with metabolism, which was hampered by changing the amino acids required for growth [12].

The present study was designed to examine the effects of cellulose on the growth performance, gut health indices, and immune responses of weaning pigs under farm-like conditions. The hypothesis tested was that the addition of cellulose in diets would not impair growth performance and gut health reared in two different sanitary conditions (good and poor) for 14 day after weaning.

## MATERIALS AND METHODS

All experimental procedures received prior approval from Animal Ethics Committee of Chungnam National University (CNU-00811).

### Animals and managements

A total of 108 male (Duroc×[Yorkshire×Landrace]) pigs were weaned at 21 day of age with the initial body weight of 6.2±0.4 kg (mean±standard error of mean). Pigs were obtained from a local government experimental farm (Chungnam Livestock Research Institute, Cheongyang-gun, Chungchung-namdo, South Korea) transported to the animal husbandry at Chungnam National University. No animal showed any signs of disease at the allocation. Six replicate pens with three pigs per pen were assigned to their experimental treatments based on initial body weight and block within a room in the animal facility. The facility contained two rooms that allowed pigs to be housed separately to avoid any cross-contamination between good sanitary and poor sanitary conditions. Each pen was equipped with a nipple bowl drinker and a metal feeder. The ambient temperature was maintained at 29°C±1°C for the initial week and then gradually decreased by 1°C every week. Pigs were offered the experimental diets and freshwater on an *ad libitum* basis.

### Experimental design and diets

This experiment was conducted as a 2×3 factorial arrangement, with respective factors being i) two environmental conditions good sanitary condition: a previously unpopulated room cleaned and disinfected; poor sanitary condition: a previously populated room without cleaning or disinfection [10], representing the experimental environment and commercial farm, respectively, and ii) three inclusion levels of cellulose (0%, 1%, and 2%).

Three experimental diets were formulated based on corn, soybean meal, dried whey, and fish meal to meet or exceed Nutrient Requirements of Swine [12] specifications and they were iso-caloric and iso-nitrogenous. The cellulose was purchased from Sigma Chemical Co. (St Louis, MO, USA). Chromium oxide (Cr_2_O_3_) 0.3% was added to each experimental diet as an indigestible marker (apparent ileal digestibility [AID], apparent total tract digestibility [ATTD]) [13]. The composition and nutrient contents of the experimental diets are presented in Table 1.

### Data collection

Body weight was individually measured at day 0, 7, and 14 of the experiment. Remaining feed was recorded on day 7 and 14 to calculate the feed intake. Average daily gain (ADG), average daily feed intake and feed conversion ratio were calculated based on collected data.

### Assessment of fecal consistency and the incidence of diarrhea

Assessment of fecal consistency and the incidence of diarrhea were carried out daily. The visual assessment of the incidence of diarrhea was conducted using the procedure described by Heo et al [14]. The fecal consistency score was assessed according to Marquardt et al [15] with a subjective score on 3-point scale ranging from 1 to 3, where 1 = well-formed; 2 = sloppy; 3 = diarrhea. Consequently, fecal samples were collected and directly stored at −20°C on 7 and 14 days after weaning for the future chemical analysis.

### Post-mortem procedure

One pig per pen (n = 36) closest to the median body weight in each pen was selected and euthanized by administering 0.1 mL of Succipharm (Komipharm Co., Ltd., Shieung, Korea) on day 0, 7, and 14. Blood samples were collected via jugular vein puncture into 5 mL vacutainer tubes coated with lithium heparin (Becton Dickinson, Franklin Lakes, NJ, USA) before pigs were euthanized. Thereafter, pigs were transferred into a chamber, where residual air was rapidly flushed with 100% carbon dioxide until death was confirmed. The abdomen was then immediately opened from the sternum to the pubis, and the gastrointestinal tract was removed, and 3 to 4 cm fragment of distal ileum was dissected from the body as described by Heo et al [16] and gently washed with pH 7.4 phosphate-buffered saline and preserved in 10% formalin solution for subsequent histological examination and immunofluorescence. Ileal digesta samples were collected from a portion of 30 cm prior to ileocecal junction to analyze apparent ileal nutrient digestibility and stored in labelled sterile containers at −20°C for further analysis.

### Chemical analyses

Samples of digesta were freeze-dried and samples of feces were heat-dried (60°C, 48 h). All the samples finely ground through a 0.5 mm screen using a grinder (Retsch Model Z-I, Stuttgart, Germany). Dry matter (DM) was determined according to the Association of Official Analytical Chemists (AOAC) method [17] (procedure # 934.01). The GE was measured using an adiabatic bomb calorimeter (model 6300, Parr Instrument, Moline, IL, USA) which has been calibrated using benzoic acid as a standard. Total nitrogen contents of samples were determined using the Dumas combustion method with a LECO CNS-2000 N analyzer (LECO Corp., St. Joseph, MI, USA) and crude protein (CP) contents of samples were calculated by multiplying the nitrogen value by 6.25. Chromium oxide levels were determined according to the procedure described by Williams et al [18] via UV absorption spectrophotometry (Ultrospec 2100 pro, Amersham Bioscience). Analyses of neutral detergent fiber, acid detergent fiber, and the determination of hemicellulose content were performed as described by Van Soest et al [19].

### Digestibility calculation

The AID and ATTD of DM, CP, and GE was calculated for each diet.

$$\text{AID or ATTD}=1-({\text{Nutrient}}_{\text{digesta}}/{\text{Nutrient}}_{\text{diet}})\times ({\text{Cr}}_{\text{diet}}/{\text{Cr}}_{\text{digesta}}),$$

AID or ATTD represent the AID and ATTD of each nutrient, respectively. Nutrient_diet_ is the amount of corresponding nutrient in the diet, and Nutrient_digesta_ is the amount corresponding same nutrient in the ileal or fecal digesta, Cr_diet_ is the Cr concentration in the diet, and Cr_digesta_ is the Cr concentration in the ileal or fecal digesta.

### Ileum morphology

The tissue preparation and methods for microscopic measurements were conducted according to the procedures described by Heo et al [16]. Briefly, after fixation in the 10% phosphate buffered saline, ring-shaped lengths of the ileal fragment were excised, dehydrated, and embedded in paraffin wax. For each of these, 6 transverse sections (4 to 6 μm) were cut, stained with haematoxylin and eosin, and mounted on glass slides. The height of 10 well-oriented villi and their associated crypts were measured with a light microscope (OLYMPUS CX31, Tokyo, Japan) using a calibrated eyepiece graticule.

### Enzyme linked immunosorbent assay measurements

The blood samples were separated by centrifugation (Micro 12, Hanil Science Co., Ltd., Incheon, South Korea) at 2,000×g for 10 min at 4°C. The supernatants, blood plasma, were transferred sterile 2 mL Eppendorf tubes. The concentrations of interleukin 1β (IL-1β; R&D Systems, Minneapolis, MN, USA), tumor necrosis factor α (TNF-α; R&D Systems, Minneapolis, MN, USA), prostaglandin E_2_ (PGE_2_; MyBioSource, San Diego, CA, USA), leukotriene B_4_ (MyBioSource, USA), and cyclooxygenase-2 (COX-2; MyBioSource, USA) in blood plasma were quantified using commercially available enzyme linked immunosorbent assay kits according to the manufacturer’s instructions.

### Immunofluorescence

Ileum tissues were fixed in 4% paraformaldehyde, washed and embedded in paraffin. Tissue sections were cut, placed on slides, de-paraffinized, rehydrated, blocked, and incubated with rabbit polyclonal anti-occludin antibody (Thermo Fisher Scientific Inc., Waltham, MA, USA) and mouse monoclonal anti-actin antibody (Abcam, Cambridge, UK) for overnight at 4°C, and then washed, and incubated with Alexa Fluor 488 conjugated goat anti-rabbit IgG secondary antibody (Thermo Fisher Scientific Inc., USA), Alexa Fluor 594 conjugated goat anti-mouse immunoglobulin G secondary antibody, and 4, 6-diamidino-2-phenylindole (Thermo Fisher Scientific Inc., USA) for 40 minutes at room temperature. The fluorescence microscope (Nikon Eclipse Ci microscope, Nikon Instruments Inc., Seoul, Korea) was used to assess the slides.

Intraepithelial tight junction integrity and the localization of occludin, transmembrane tight junction protein was examined to determine the influence of environmental conditions and dietary treatments. The occludin localization was determined using a three-scale visual assessment (score 1 = occludin was localized to the apical intercellular region of ileal epithelium; score 2 = occludin was partly localized to the apical intercellular region while some occludin was diffused at the intercellular tight junction region; score 3 = most of the occludin was diffused at the intercellular tight junction region. Collected data were then expressed as the proportion of occludin diffused at the ileal epithelium. A higher diffusion score indicated more disruption of the intraepithelial tight junction.

### Statistical analyses

Data were analyzed as a completely randomized block design, using the general linear model procedure of IBM SPSS Statistics 22 (IBM SPSS, Chicago, IL, USA). The individual pig was the experimental unit for all measures except growth performance, and the model included dietary treatments and environmental conditions as two main effects. Pigs were blocked based on the body weight at weaning, and the block was used as a random factor in the model for all variables. Body weight, ileum morphology and blood measurements on day 0 were included in the model as a covariate for analyses of growth performance, ileum morphology and cytokine concentrations in the blood, respectively. Data for the incidence of post-weaning diarrhea were expressed as the mean percentage of days with diarrhea relative to the 14 day after weaning [14]. The statistical significance was accepted at p< 0.05, and the trend was considered at p<0.10 Tukey’s multiple range test was used to separate the means of treatments where appropriate.

## RESULTS

No mortality was observed in response to the different environmental conditions or supplementation with cellulose in the diets over the entire experimental period.

### Growth performance

No interaction between the environmental conditions and the inclusion of cellulose to the diet was observed on growth performance from day 1 to 14 (Table 2). Pigs exposed to the poor sanitary conditions had lower ADG than those pigs housed in the good sanitary conditions from day 1 to 7 (p< 0.001). However, the pigs were not affected (p>0.05) by the cellulose supplementation or environmental conditions from day 7 to 14.

### Intestinal morphology

The effects of environmental conditions and the addition of cellulose to the diet on ileal morphology on day 7 and 14 are presented in Table 3. On day 7, pigs housed under sanitary conditions exhibited healthier ileal villous height (p<0.001) and crypt depth (p<0.001) than those housed under poor sanitary conditions. The addition of 1% cellulose to the diet reduced the ileal crypt depth in pigs housed under good sanitary conditions (p<0.05). Interactions between environmental conditions and the addition of cellulose to the diet were observed on villous height (p<0.10), crypt depth (p< 0.01), and villous to crypt ratio (p<0.001) in the ileum. The supplementation of 2% cellulose to the diet showed the low crypt depth (p<0.01) in the poor sanitary conditions. Between the poor sanitary conditions and good sanitary conditions, the supplementation of 1% cellulose to the diet showed the opposite results.

On day 14, pigs exposed to the poor sanitary conditions displayed a shorter villous height (p<0.001) and deeper crypt depth (p<0.001) compared to those housed under good sanitary conditions. The pigs that received a diet supplemented with 1% cellulose had a lower crypt depth (p< 0.001) and improved villous to crypt ratio (p<0.01) compared to their counterparts regardless of the environmental conditions. Interactions between environmental conditions and the addition of cellulose to the diet were observed on crypt depth (p<0.001) and villous height to crypt depth ratio (p<0.01) in the ileum. Weaning pigs under the good sanitary condition that received the diet without cellulose showed a deeper crypt depth (p<0.05) and lower villous height to crypt depth ratio (p<0.05) compared to those fed diets supplemented dietary cellulose. However, pigs fed a diet with 2% cellulose demonstrated a deeper crypt depth (p<0.05) and lower villous height to crypt depth ratio compared to their counterparts under the poor sanitary conditions.

### The incidence of diarrhea and occludin localization

The effects of environmental conditions and the addition of cellulose to the diet on the incidence of diarrhea are presented in Table 4. No interaction (p>0.05) between environmental conditions and the addition of cellulose to the diet was observed throughout the experimental period. From day1 to 14, poor sanitary conditions increased the incidence of diarrhea regardless of the cellulose in the diet (p<0.05).

The proportion of occludin diffused at the ileal epithelium is presented in Table 5. The occludin diffusion score was not affected (p>0.05) by environmental conditions or by the addition of cellulose in the diet, and thus no interaction observed (p>0.05) due to those factors.

### Ileal and total tract digestibility

Apparent ileal and total tract digestibility of nutrients on day 7 and 14 are presented in Tables 6 and 7, respectively.

No interactions (p>0.05) between the environmental conditions and dietary treatments were observed on day 7 and 14 for ileal and total tract digestibility of nutrients.

On day 7, pigs housed under poor sanitary conditions decreased their ileal digestibility of GE compared to those housed under good sanitary conditions (p<0.05). The addition of cellulose 2% to the diet decreased the apparent ileal digestibility (p<0.05) of DM (p<0.05), CP (p<0.10), and GE (p<0.10). Similarly, the total tract digestibility of DM was decreased (p<0.05) when the pigs were fed the diets supplemented with 1% and 2% cellulose. The total tract digestibility of CP tended to decrease when additional cellulose was supplemented to the diet (p<0.10). The total tract digestibility of GE was not altered (p>0.05) by environmental conditions or by the addition of cellulose to the diet.

On day 14, the poor sanitary conditions decreased (p <0.05) the ileal DM digestibility in pigs regardless of the cellulose inclusion in the diet. The inclusion of cellulose 2% decreased (p<0.05) the ileal CP digestibility in pigs regardless of the environmental conditions. The ileal GE digestibility was not affected (p>0.05) by the environmental conditions and the addition of cellulose to the diet. Subsequently, pigs exposed to the poor sanitary conditions had lower total tract digestibility of DM (p<0.05) and CP (p<0.05), as well as GE (p<0.10). The addition of cellulose to the diet decreased the total tract digestibility of CP (p<0.05), GE (p<0.05), and total tract DM digestibility (p<0.10).

### Circulation of cytokines and lipid mediators

The interaction between environmental condition and cellulose levels did not show any statistical differences (p>0.05) for the circulation of cytokines and lipid mediators (Table 8).

On day 7, pigs housed under the poor sanitary conditions showed increased TNF-α (p<0.10). COX-2 and PGE_2_ also increased when the pigs were exposed to poor sanitary conditions (p<0.05). However, circulating COX-2 decreased when the pigs were fed a diet supplemented with 1% and 2% cellulose compared to those fed a diet without the addition of cellulose.

On day 14, a poor sanitary condition increased COX-2 and PGE_2_ (p<0.05) in pigs and increased TNF-α (p<0.10). However, the addition of cellulose in the diet did not alter the pro-inflammatory cytokines and lipid mediators on day 14.

## DISCUSSION

In the present study, we hypothesized that pigs fed a diet supplemented with dietary cellulose would decrease their pro-inframammary responses by reducing the incidence of post-weaning diarrhea due to the induced environmental challenge, thus leading to improved growth performance for 14 day after weaning. During the critical period (14 day after weaning), there are many assumptions that young pigs exposed to a poor environmental condition due to the weaning transition together with drastic changes in physical and mental conditions (e.g., depleted autoimmunity and mixture of litter mates) can lead pigs to encounter intestinal dysfunction and enteric pathogen-associated diarrhea, in turn experiencing growth-check of weaning pigs [1,8]. In the present study, a pure form of cellulose was utilized as a source of insoluble non-starch polysaccharide (NSP) that can be easily included in commercial diets, and thus is worthy of testing its function in young pigs [20]. Nonetheless, its influence on the host animals is highly variable because NSP affects the digesta flow rate, feed intake, and growth [21]. We chose an environmental challenge model to mimic a commercial setting, where pigs were exposed to numerous biological impacts, and thus their responses are unlike those of the sanitary conditions of an experimental research facility [6].

Our data demonstrated that an environmental challenge decreased the growth performance of young pigs. In our results, there was a pig observed a negative ADG due to environmental challenge during 7 days after weaning. However, those pigs fed a diet supplemented with cellulose did not decrease their ADG, independent of the concentrations added and the facility conditions. Pascoal et al [4] reported that feeding a diet supplemented with 1.5% purified cellulose did not hinder growth performance of weaned pigs. Although we did not measure bacterial diversity, a linear homopolymer of β-(1→4) linked glucose units of insoluble dietary fiber (cellulose) could result in a faster transit time of chyme, and subsequently, a reduced time available for the adhesion of pathogenic bacteria in the gastrointestinal tract of young pigs. Dietary cellulose could possibly improve intestinal morphology and nutrient digestibility (Tables 3, 6) [22]. The environmental challenge decreased growth performance of young pigs, indicating that such a model was successful in the present study. The pigs housed under the poor environmental conditions that had lower growth performance could be due to the higher incidence of diarrhea, lower nutrient digestibility, and retrogression of villous/crypt ratio of young pigs [1,14].

In the present study, the nutrient digestibility decreased when the pigs were fed higher dietary cellulose. This finding is following the study by Gutierrez et al [23], who reported a reduction in nutrient digestibility of DM, CP, and GE when the concentration of insoluble dietary fiber was increased in pigs. This could be due to insoluble dietary fiber stimulates the peristaltic action and decreases the residence time of digesta in the gastrointestinal tract, and thus impairs nutrient utilization [24]. Similarly, Son and Kim [25] found that growing pigs fed a diet supplemented with microcrystalline cellulose resulted in the reduction of nutrient digestibility by increasing digesta flow rate. However, there was no contribution to the overall growth performance of pigs fed the basal diet that had higher digestibility. In addition, we were not unfortunately in position to measure the flow rate of digesta in the intestinal tract.

The tight junction where the side faces toward the inside of the intestine plays an important role in the regulation of gut permeability by preventing the invasion of bacteria, viruses, and endotoxins. The occludin, the integral transmembrane-associated and tight junction-associated protein, has been characterized as localizing the tight junction system of fibrils in the gut epithelium and forming the solid tight junction system. Therefore, post-weaning diarrhea can be partially explained by the immunofluorescence staining of horizontal sections of the intestine revealing a configuration of occludin [26]. The noticeable depletion of occludin is induced by bacterial and viral infection. In the present study, however, the occludin localization in the ileum was not affected by environmental conditions together with the cellulose inclusion levels. It is assumed that the environmental challenge conducted in the present study did not compromise the tight junction structure in terms of unaltered localization of occludin in the ileum and this assumption linked to the insignificant difference in the diarrhea index. However, the function and quantification of occludin remain elusive and further research is required to determine whether severe challenge models such as the injection of lipopolysaccharides to damage the intestinal tight junction is correlated with the occludin localization.

One of the detrimental consequences that occur during weaning is alterations in pro-inflammatory responses, which has a great influence on growth performance and immunity in pigs. The ongoing immune and inflammatory responses along the pig gastrointestinal tract cost a significant amount of nutrients and compete with nutrients for growth. Shin et al [27] reported that weaned pigs challenged with unsanitary farm conditions showed increased blood levels of pro-inflammatory cytokine IL-1β and eicosanoid PGE_2_, with these increments resulting from the poor sanitary housing as well as the induced secondary inflammation followed by the weaning process. Other previous studies have revealed that upregulated pro-inflammatory cytokines are particularly associated with the decreased nutrient digestibility due to the impaired epithelial functions related to the transportation of nutrients and ions [28]. Pié et al [28] reported that upregulated TNF-α expression was observed one day after weaning and kept increasing up to eight days after weaning. Our findings are in accordance with this study that reported a higher concentration of pro-inflammatory cytokines and lipid mediators in the blood on day 7 compared to day 14 after weaning regardless of environmental conditions. Therefore, the continuous weaning stress might contribute to increased blood TNF-α, COX-2, and PGE_2_ on both day 7 and 14 after weaning and the combined effects of weaning stress and poor sanitary conditions were more detrimental in pigs. The blood COX-2 levels were only affected by cellulose inclusion on day 7 after weaning. COX-2 modulates prostaglandin secretion, which is important in the alleviation of mucosal injury, protection against bacterial invasion, and downregulation of the mucosal immune system [29].

Our results showed that young pigs fed the diet supplemented with cellulose increased villous height and decreased crypt depth, indicating an improvement in the health of the small intestine. This result is in accordance with previous results demonstrated by, Montagne et al [10] showed that pigs fed diets with insoluble NSP (e.g., cellulose) had higher villous and shorter crypt depth under poor sanitary conditions. Therefore, feeding insoluble NSP could help pigs cope with a viable environment with beneficial intestinal bacteria. There could be a complex link between housing conditions and insoluble dietary fiber supplementation to young pigs; however, the prebiotic functions of dietary fiber observed even under disease challenge models indicate that insoluble dietary fiber may be an effective additive in the diet of weaned pigs [30]. In contrast, Pelicano et al [31] suggested that intestinal villous height was increased when Bacillus subtilis was associated with prebiotics.

## CONCLUSION

Our results indicated that the under poor sanitary condition observed negative effects of growth performance, ileal morphology, diarrhea index, digestibility and immunity system for 14 day after weaning. Cellulose supplementation impaired nutrient digestibility irrespective of environmental conditions. Nevertheless, the diet supplemented with cellulose would be considered as a strategy to increase villous height to crypt depth ratio together with decreased COX-2 suggesting healthier of the gastrointestinal tract in pigs for 14 days after weaning.

## Figures and Tables

**Table 1:** Composition of the experimental diet (as-fed basis)

Item	Cellulose 0%	Cellulose 1%	Cellulose 2%
Ingredients (%)			
Corn	46.34	45.34	44.34
Soybean meal, 44% crude protein	16.00	16.00	16.00
Dried whey	15.00	15.00	15.00
Fish meal	11.46	11.46	11.46
Spray-dried plasma	4.00	4.00	4.00
Lactose	3.00	3.00	3.00
Soybean oil	3.00	3.00	3.00
Limestone	0.50	0.50	0.50
Cellulose^1)^	-	1.00	2.00
Chromium oxide	0.30	0.30	0.30
Vitamin-Mineral premix^2)^	0.40	0.40	0.40
Calculated chemical composition			
Metabolizable energy (MJ/kg)	14.81	14.81	14.81
Crude protein (%)	23.1	23.1	23.1
Lysine (%)	1.5	1.5	1.5
Analyzed composition			
Gross energy (MJ/kg)	16.39	16.26	16.49
Crude protein (%)	23.28	23.01	23.13
Neutral detergent fiber (%)	6.9	7.4	7.6
Acid detergent fiber (%)	2.1	2.3	2.5

1) Accent Microcell Pvt. Ltd (SATELLITE, Ahmedabad-380015, Gujarat, India).

2) Provided the following nutrients (per kg of air-dry diet): vit A 7,000 IU; vit D_3_ 1,400 IU; vit E 20 mg; vit K 1 mg; vit B_1_ 1 mg; vit B_2_ 3 mg; vit B_6_ 1.5 mg; vit B_12_ 15 μg; calcium pantothenate 10.7 mg, folic acid 0.2 mg, niacin 12 mg, biotin 30 μg. Minerals: Co 0.2 mg (as cobalt sulphate); Cu 10 mg (as copper sulphate); iodine 0.5 mg (as potassium iodine); iron 60 mg (as ferrous sulphate); Mn 40 mg (as manganous oxide); Se 0.3 mg (as sodium selenite); Zn 100 mg (as zinc oxide).

**Table 2:** Effects of environmental conditions and cellulose levels on growth performance in weaned pigs

Item	Feeding regimen						SEM	p-value^1)^		

	Good sanitary			Poor sanitary						
		
	Cellulose 0%	Cellulose 1%	Cellulose 2%	Cellulose 0%	Cellulose 1%	Cellulose 2%		E^2)^	D^2)^	E×D^2)^
d 1 to 7										
ADG (g)	271.17	175.66	193.09	−13.73	0.65	−22.71	21.479	^***^	NS	NS
ADFI (g)	271.50	243.74	248.79	201.04	215.99	179.94	23.403	NS	NS	NS
FCR^3)^	-	-	-	-	-	-	-	-	-	-
d 8 to 14										
ADG (g)	462.30	501.39	434.57	400.62	339.89	301.08	26.225	NS	NS	NS
ADFI (g)	574.36	554.94	463.71	481.33	473.26	355.43	33.535	NS	NS	NS
FCR	1.30	1.10	1.05	1.21	1.44	1.20	0.071	NS	NS	NS
d 1 to 14										
ADG (g)	366.73	338.53	313.83	193.45	170.27	139.19	20.934	^**^	NS	NS
ADFI (g)	422.93	399.34	356.25	341.19	344.62	267.68	26.992	NS	NS	NS
FCR	1.13	1.19	1.12	1.76	2.52	1.99	0.183	^*^	NS	NS

Values are means of 6 replicates.

SEM, pooled standard error of mean; ADG, average daily gain; ADFI, average daily feed intake; FCR, feed conversion ratio.

1) Significance level: no significance (NS),

* p<0.05,

** p<0.01,

*** p<0.001.

2) E, environmental conditions (good sanitary vs poor sanitary); D, dietary treatments; E×D, interaction between environmental conditions and dietary treatments.

3) Negative FCR values occurred for week 1 after weaning, and data were removed.

**Table 3:** Effects of environmental conditions and cellulose levels on ileal morphology in weaned pigs

Item	Feeding regimen						SEM	p-value^1)^		

	Good sanitary			Poor sanitary						
		
	Cellulose 0%	Cellulose 1%	Cellulose 2%	Cellulose 0%	Cellulose 1%	Cellulose 2%		E^2)^	D^2)^	E×D^2)^
d 7										
Villous height (μm)	712.54	754.44	753.10	556.10	510.91	579.49	7.719	^***^	NS	^†^
Crypt depth (μm)	624.31^a^	578.59^b^	665.46^a^	479.27^c^	458.45^d^	451.55^cd^	5.815	^***^	^*^	^**^
V:C6	1.17	1.37	1.16	1.24	1.17	1.29	0.019	NS	NS	^***^
d 14										
Villous height (μm)	831.09	798.79	788.81	704.32	718.79	687.63	9.141	^***^	NS	NS
Crypt depth (μm)	750.09^a^	589.39^c^	606.54^b^	554.14^cd^	531.73^d^	599.78^c^	7.868	^***^	^***^	^***^
V:C	1.15^c^	1.41^a^	1.34^b^	1.36^b^	1.40^a^	1.19^c^	0.020	NS	^**^	^**^

SEM, pooled standard error of mean; V:C, villous height: crypt depth.

1) Significance level: NS, no significance;

† p<0.10;

* p<0.05;

** p<0.01;

*** p<0.001.

2) E, environmental conditions (good sanitary vs poor sanitary); D, dietary treatments; E×D, interaction between environmental conditions and dietary treatments.

a–d Means in the same row with different superscripts differ in D (p<0.05).

**Table 4:** Effects of environmental conditions and cellulose levels on diarrhoea index in weaned pigs

Item	Feeding regimen						SEM	p-value^1)^		

	Good sanitary			Poor sanitary						
		
	Cellulose 0%	Cellulose 1%	Cellulose 2%	Cellulose 0%	Cellulose 1%	Cellulose 2%		E^2)^	D^2)^	E×D^2)^
d 7										
Diarrhea index (%)	21.43	14.29	9.52	31.43	26.79	17.86	3.060	NS	NS	NS
d 14										
Diarrhea index (%)	17.86	8.93	4.76	28.57	21.43	17.86	3.684	NS	NS	NS

The diarrhoea index was expressed as a proportion of days with diarrhoea with respect to total number of days (for 14 days).

SEM, pooled standard error of mean.

1) Significance level: NS, no significance.

2) E, environmental conditions (good sanitary vs poor sanitary); D, dietary treatments; E×D, interaction between environmental conditions and dietary treatments.

**Table 5:** Effects of environmental conditions and cellulose levels on occludin localization in weaned pig

Item	Feeding regimen						SEM	p-value^1)^		

	Good sanitary			Poor sanitary						
		
	Cellulose 0%	Cellulose 1%	Cellulose 2%	Cellulose 0%	Cellulose 1%	Cellulose 2%		E^2)^	D^2)^	E×D^2)^
d 7										
Occludin localization	40.60	43.40	56.63	57.47	72.65	64.78	7.180	NS	NS	NS
d 14										
Occludin localization	62.42	47.55	53.91	43.24	77.69	60.90	6.673	NS	NS	NS

Occludin localization criteria was expressed as the mean occludin localization (diffusion) value of pigs having score 1 (30.3%), score 2 (60.6%), and score 3 (99.9%).

SEM, pooled standard error of mean.

1) Significance level: NS, no significance.

2) E, environmental conditions (good sanitary vs poor sanitary); D, dietary treatments; E×D, interaction between environmental conditions and dietary treatments.

**Table 6:** Effects of environmental conditions and cellulose levels on apparent ileal digestibility for weaned pig for 14 days after weaning

Item	Feeding regimen						SEM	p-value^1)^		

	Good sanitary			Poor sanitary						
		
	Cellulose 0%	Cellulose 1%	Cellulose 2%	Cellulose 0%	Cellulose 1%	Cellulose 2%		E^2)^	D^2)^	E×D^2)^
d 7										
Dry matter (%)	79.30^a^	74.53^b^	71.34^c^	77.30^a^	74.32^b^	68.53^d^	1.004	NS	^*^	NS
Crude protein (%)	75.48^a^	75.35^a^	68.02^b^	75.00^a^	68.18^b^	67.70^bc^	1.044	NS	^†^	NS
Gross energy (%)	77.60^a^	76.66^ab^	73.56^c^	75.47^b^	72.08^c^	66.90^d^	1.009	^*^	^†^	NS
d 14										
Dry matter (%)	76.93	73.52	71.54	71.93	70.78	70.67	0.544	^*^	NS	NS
Crude protein (%)	76.56^a^	73.50^bc^	66.26^d^	77.89^a^	75.33^b^	71.25^c^	1.062	NS	^*^	NS
Gross energy (%)	76.70	75.20	70.53	76.92	79.74	76.37	1.153	NS	NS	NS

SEM, pooled standard error of mean.

1) Significance level: NS, no significance;

† p<0.10;

* p<0.05.

2) E, environmental conditions (good sanitary vs poor sanitary); D, dietary treatments; E×D, interaction between environmental conditions and dietary treatments.

a–d Means in the same row with different superscripts differ in D (p<0.10).

**Table 7:** Effects of environmental condition and cellulose levels on apparent total tract digestibility for weaned pig for 14 days after weaning

Item	Feeding regimen						SEM	p-value^1)^		

	Good sanitary			Poor sanitary						
		
	Cellulose 0%	Cellulose 1%	Cellulose 2%	Cellulose 0%	Cellulose 1%	Cellulose 2%		E^2)^	D^2)^	E×D^2)^
d 7 (%)										
Dry matter	87.06^a^	83.40^b^	80.52^c^	86.64^a^	78.54^d^	77.59^d^	0.940	NS	^*^	NS
Crude protein	84.35^a^	83.28^a^	74.94^d^	82.75^b^	78.31^c^	77.19^c^	1.100	NS	^†^	NS
Gross energy	84.51	84.40	78.04	84.25	81.40	80.71	1.046	NS	NS	NS
d 14 (%)										
Dry matter	87.50^a^	85.76^b^	81.64^c^	86.39^ab^	79.58^cd^	77.61^d^	0.911	^*^	^†^	NS
Crude protein	87.13^a^	84.75^b^	79.35^c^	83.13^b^	80.01^c^	70.23^d^	1.334	^*^	^*^	NS
Gross energy	88.77^a^	86.27^b^	81.96^d^	83.66^c^	81.01^d^	74.86^e^	1.155	^†^	^*^	NS

SEM, pooled standard error of mean.

1) Significance level: NS, no significance;

† p<0.10,

* p<0.05.

2) E, environmental conditions (good sanitary vs poor sanitary); D, dietary treatments; E×D, interaction between environmental conditions and dietary treatments.

a–e Means in the same row with different superscripts differ in D (p<0.10).

**Table 8:** Effects of environmental conditions and cellulose levels on pro-inflammatory cytokines and lipid mediators for weaned pig for 14 days after weaning

Item	Feeding regimen						SEM	p-value^1)^		

	Good sanitary			Poor sanitary						
		
	Cellulose 0%	Cellulose 1%	Cellulose 2%	Cellulose 0%	Cellulose 1%	Cellulose 2%		E^2)^	D^2)^	E×D^2)^
d 7 (pg/mL)										
IL-1β	0.00	0.00	0.00	0.00	12.00	0.00	1.262	NS	NS	NS
TNF-α	9.66	0.97	2.68	26.35	18.24	9.23	3.637	^†^	NS	NS
COX-2	109.81^b^	100.78^c^	84.00^d^	133.70^a^	116.80^b^	99.13^c^	3.209	^*^	^*^	NS
PGE_2_	6.60	12.56	12.82	36.19	67.52	28.38	5.022	^*^	NS	NS
LTB_4_	452.77	307.70	90.79	785.46	567.55	160.91	66.402	NS	NS	NS
d 14 (pg/mL)										
IL-1β	0.00	0.00	0.00	0.00	67.69	0.00	7.574	NS	NS	NS
TNF-α	6.34	5.01	5.86	25.23	18.83	7.94	2.857	^†^	NS	NS
COX-2	116.08	98.56	107.85	141.62	133.29	117.38	5.041	^*^	NS	NS
PGE_2_	8.17	27.19	37.59	103.01	55.78	70.79	7.977	^*^	NS	NS
LTB_4_	277.06	218.54	201.13	417.64	375.02	175.56	64.075	NS	NS	NS

SEM, pooled standard error of mean; IL-1β, interleukin 1β; TNF-α, tumor necrosis factor α; COX-2, cyclooxygenase-2; PGE_2_, prostaglandin E_2_; LTB_4_, leukotriene B_4_.

1) Significance level: NS, no significance;

† p<0.10,

* p<0.05.

2) E, environmental conditions (good sanitary vs poor sanitary); D, dietary treatments; E×D, interaction between environmental conditions and dietary treatments.

a–d Means in the same row with different superscripts differ in D (p<0.05).
